# Comparison of two column agglutination tests for red blood cell antibody testing

**DOI:** 10.1371/journal.pone.0210099

**Published:** 2018-12-31

**Authors:** Jonas Sawierucha, Marion Posset, Viola Hähnel, Christian L. Johnson, James A. Hutchinson, Norbert Ahrens

**Affiliations:** 1 Institute for Clinical Chemistry and Laboratory Medicine, Transfusion Medicine, University Hospital Regensburg, Regensburg, Germany; 2 Department of Surgery, University Hospital Regensburg, Regensburg, Germany; The Ohio State University, UNITED STATES

## Abstract

**Background:**

Several sensitive methods are available for red blood cell (RBC) antibody screening. Among these, gel and glass card systems have demonstrated comparably good performance in retrospective studies and are widely used in routine patient diagnostics, but their performance in prospective studies has not been sufficiently characterised.

**Patients and methods:**

Gel card (Bio-Rad DiaMed) and glass bead-based (Ortho Clinical Diagnostics) column agglutination technologies were used to screen for antibodies prospectively (group A) and for antibody identification in stored and fresh samples known to contain RBC antibodies retrospectively (group B). Untreated reagent RBCs and either papain-treated (Bio-Rad) or ficin-treated panel C cells (Ortho) were used for antibody identification.

**Results:**

RBC-reactive antibodies were detected in 22 of 1000 group A samples, three of which tested positive only by gel card agglutination, and four only by glass bead agglutination (including one false positive each). Group B comprised 202 sera with known antibodies: 33 of these samples contained 36 antibodies detected only by gel card agglutination, whereas 9 samples contained antibodies detectable only by glass bead-based agglutination. Discrepancies mostly involved weak antibodies reactive by enzyme only. Two sera contained antibody mixtures that neither system detected completely. Of note, in antibody differentiation batches one and two, anti-Lu^a^ was reactive in 7 of 7 and 1 of 8 samples, respectively.

**Conclusion:**

Both column agglutination tests for red cell antibodies had equal sensitivity and specificity with unstored samples. In stored samples, weak and enzyme-only antibodies were more frequently detected with the gel card system.

## Introduction

Red blood cell (RBC) immunization is one of the most frequent side effects of blood transfusion. Up to 8% of RBC recipients may develop new antibodies after a single transfusion episode [1]. Patients that develop alloantibodies are also at risk to develop autoimmunization against RBC antigens. In fact, alloimmunization is the most frequent cause for autoimmunization [2]. As about half to two third of the alloantibodies become undetectable within four to five years [3–5], the affected patients are at risk of developing delayed serologic or hemolytic transfusion reactions after subsequent blood transfusions. Therefore, RBC antibodies are among the most common causes of transfusion morbidity [6, 7]. RBC antibodies were also the most frequent transfusion cause of death accounting for 10 out of 25 cases in a recent report of the European Commission [8]. Sensitive detection of these antibodies is essential.

Several techniques have been developed for this purpose. Low Ionic Strength Solution (LISS)-Antihuman Globulin, polyethylene glycol and polybrene have been introduced as reaction enhancers, and antigen-antibody reactions were transferred from tube to column agglutination or microplate solid-phase reactions. Among these, antihuman globulin non-tube tests have been found to be superior to the others [9]. Solid-phase reactions on microtiter plates [10] are known to be sensitive but lack sufficient specificity: They produce inconclusive results 2.5 to 10 times more frequently than column systems [11, 12]. Column agglutination may be performed using cards that either trap agglutinates in a dextran-acrylamide matrix, or in a matrix consisting of glass beads [13–15]. These similar methods have been investigated with stored samples, blood donors or in house reagent cells. Data on the performance in patient diagnostics is incomplete.

Therefore, we compared gel with glass bead based column agglutination technologies. Our results indicate that both systems have equal sensitivity and specificity in screening. However, we observed differences in identification related to the setup of the panels.

## Material and methods

### Reagents

RBC antibody screening and identification tests from Bio-Rad DiaMed (Cressier-sur-Morat, Switzerland; gel card) [13] and Ortho Clinical Diagnostics (Raritan, New Jersey, USA; glass card) [14] were used according to the manufacturer’s instructions. Reaction strength was graded on a scale of zero (negative) to four-plus (complete agglutination). Screening was performed using a panel of three reagent cells in antihuman globulin without enzyme-treated cells (ID-DIA Panel I-II-III test cells, Surgiscreen test cells), which homozygously expressed the antigens D, c, Fy^a^, Fy^b^, Jk^a^, Jk^b^, S, and s as well as C, E, e, C^W^, K, k, Kp^a^, Kp^b^, Le^a^, Le^b^, M, N, Lu^a^, Lu^b^, and P_1_. Antibody identification with both systems was performed using untreated and either papain-treated (DiaMed) or ficin-treated (Ortho) reagent cells (ID-Dia Panel or Resolve Panel C). Untreated reagent cells were tested in antihuman globulin (LISS/Coombs gel cards, BioVue System Poly Cassettes), and enzyme-treated cells in a saline milieu (neutral gel card, BioVue neutral cassettes). Gel card samples were pipetted manually, and glass card samples were pipetted using an automated platform (AutoVue Innova). Reactions were classified as specifically positive if at least homozygous cells were weakly reactive (+/–).

### Patients and samples

Two groups of samples were investigated as approved by the local ethics committee (Independent Ethics Committee at the University of Regensburg, 17-537-101). Group A consisted of 1000 unstored local routine EDTA-anticoagulated samples that were recruited from local patients of the University Hospital Regensburg with a case mix index of 2.0 and 835 beds that are mainly focused on intensive care with approximately 28,000 antibody screens and 18,000 red cell transfusions annually. These were screened for antibodies prospectively and in parallel (by both test systems) from the end of March to the end of April 2017. If a sample tested positive by one or both methods, antibody identification was performed by both systems.

Group B consisted of 202 sera with known antibodies that were mainly collected locally as well as from cooperating blood banks. Antibody identification of these samples was performed retrospectively and in parallel from the end of March to the middle of August 2017. RBC screening was not performed in group B.

## Results

Prospective antibody screening was performed in a total of 1000 samples in group A (Table 1) using both gel cards and glass bead cards. Irregular antibodies were detected in a total of 22 samples (2.2%) from 17 group A patients (2.5%), seven samples of which had more than one antibody (32%).

**Table 1:** Patient and sample characteristics.

	Group A	Group B
Patients (n)	687	180
Patient age* (years)	60 (range: 1–94)	68 (range: 11–92)
Samples (n)	1000	202

*Data expressed as median with range in parenthesis

Both test systems yielded true positive results for 17 samples containing mostly clinically relevant antibodies (Table 2). A further 3 samples only tested positive by gel card (including one false positive), whereas additional 4 samples were only positive by glass card (also including one false positive, Table 3 and Table 4). Samples were regarded as false positive if positive screening could not be confirmed by differentiation (ie. sporadic reaction without determinable antigen specificity). Altogether, reaction discrepancies were observed in 7 of 24 samples (29%), all of which had weak reactions graded one-plus or lower (Table 4). This included one sample with a true positive glass card reaction and a false negative gel card reaction: antibody differentiation was possible in the case of the gel card system but not the glass card system. In group A, the difference between the two systems was not significant (in McNemar’s test, the p-value was 1).

**Table 2:** Concordant positive RBC antibody test results.

Group	Identification results	Total no. of samples and relevance
A	D, C, E, WAA	1, relevant
A	Fy^a^	2, relevant
A	D	2, relevant
A	Lu^a^	3, relevant
A	Cw	1, relevant
A	C, D	3, relevant
A	M	1, relevant
A	D, E	1, relevant
A	D, Jk^a^	1, relevant
A	Daratumumab	2, irrelevant

WAA, warm-reactive autoantibodies

**Table 3:** RBC antibody test results in Group A (prospective screening samples) versus Group B (stored samples).

	Group A		Group B	
	Gel card	Glass card	Gel card	Glass card
Samples with correct positive test	19	20	191	167
Samples with incorrect positive test	1	1	0	0
Samples with correct negative test	977	977	0	0
Samples with incorrect negative or incomplete test	3	2	11	35
Accuracy	99.6%	99.7%	NaN	NaN
Sensitivity	86.36%	90.90%	NaN	NaN
Specificity	99.90%	99.90%	NaN	NaN
PPV	95.00%	95.24%	94.55%	82.67%
NPV	99.69%	99.80%	NaN	NaN

Accuracy calculated as (true positives + true negatives) / all samples; NaN, not a number; NPV, negative predictive value calculated as true negatives / (true negatives + false negatives); PPV, positive predictive value calculated as true positives / (true positives + false positives); sensitivity calculated as true positives / (true positives + false negatives); specificity calculated as true negatives / (true negatives + false positives)

**Table 4:** Discordant RBC antibody test investigation results.

Type of discrepancy	Group	Gel card		Glass card		Total no. of samples and relevance of discrepancy
		screening	identification	screening	identification	
Glass card positive, gel card initially negative or incomplete	A	Negative	Negative	Positive	Lu^a^	1, relevant
	A	Negative	K	Positive	Negative	1, relevant
	A	Negative	WAA	Positive	WAA	1, irrelevant
	A	Negative	Negative	Positive	No antibody specificity*	1, irrelevant
	B		Negative		K (n = 2); Jk^a^; Lu^a^;	4, relevant
	B		Negative		N; P_1_	2, irrelevant
	B		E		E + C	1, relevant
	B		Cw		Cw + Jk^a^	1, relevant
	B		Jk^a^		Jk^a^ + WAA	1, irrelevant
Gel card positive, glass card initially negative or incomplete	A	Positive	D + E	Negative	D + E	1, relevant
	A	Positive	WAA	Negative	WAA	1, irrelevant
	A	Positive	Negative	Negative	Negative	1, irrelevant
	B		M		No antibody specificity *	1, relevant
	B		Le^b^		No antibody specificity *	1, irrelevant
	B		M; Jk^a^; Le^a^; C; Lu^a^ (n = 4); E + M; Jk^a^ + Lu^a^		Negative	10, relevant
	B		D + C		D	6, relevant
	B		C + WAA		C	1, irrelevant
	B		E + WAA		E	1, irrelevant
	B		D + C + WAA		D + C	2, irrelevant
	B		D + C + E		D + E	4, relevant
	B		D + E		D (n = 2); E	3, relevant
	B		D + C + E + WAA		D + E	1, relevant
	B		D + C + E + WAA		D + C + E	1, irrelevant
	B		D + C +Jk^a^ + WAA		D + Jk^a^ + WAA	1, relevant
	B		D + C + Lu^a^		D + C	1, relevant
Both incomplete	B		E		Lu^a^	1, relevant
	B		Lu^a^		WAA	1, relevant

WAA, warm-reactive autoantibodies

*sporadic reactions

Group B samples consisted of 202 known positive samples from 180 patients with a total of 327 antibodies. 82 (41%) of these samples contained more than one antibody (including autoantibodies). As group B included only those sera that were reactive upon retesting, sensitivity, specificity, and accuracy could not be determined for that group. In fact, a further 10 samples were non-reactive after storage at –30°C for up to 5 years and therefore not included.

Both systems were positive for 158 of the 202 antibody-containing group B samples, including two samples with incomplete test results with both systems (Table 4). Altogether, gel card testing produced false negative results for 11 samples, and glass card testing yielded false negative results for 35 samples.

There were discrepancies between results for certain blood groups. In group B samples, antibodies to Rh antigens appeared to be more reactive in the gel card system. Of the 53 group B sera containing anti-C, 40 were reactive in glass card testing compared to 52 in gel card testing. Of a total of 123 samples containing antibodies against Rh antigens, 106 were detected by the glass card system compared to 122 by the gel card system. Warm-reactive autoantibodies (WAA), which usually display some Rh reactivity, were detected in 18 of 24 samples (glass card) versus 22 of 24 samples (gel card). This difference indicates that reagent cells were more intensely treated by the gel card system’s enzyme (papain) than by that of the glass card system (ficin) and, thus, were more reactive to antibodies to the Rh system.

Autoantibodies were present in 26 samples: 18 were detected by both systems; six by gel card only; and two by glass card only. This finding is consistent with the more intense enzyme treatment of reagent cells associated with the gel card system.

No difference between systems was observed for antibodies to Jk antigens. A total of 13 group B sera contained anti-Jk^a^: nine were detected by both systems, two by gel card testing only, and another two by glass card testing only.

Antibodies to the Kell system were present in 22 group B samples: The glass card system detected all twenty-two while the gel card system detected twenty.

In group B, 44 of 202 samples (22%) gave discrepant results using the two assays (Table 4) and this difference was significant (p < 0.005 in McNemar’s test). This included seven samples containing anti-Lu^a^, which was not detected or was incompletely recognized in glass card testing. All of these failed in the latter of two reagent cell batches, in which only one additional anti-Lu^a^ was recognized. The newer identification batch was Lu^a^-positive for one of eleven cells. In contrast, the previous batch (which was also used for group A identification) recognized all of seven previously tested group B samples that contained anti-Lu^a^. This previous batch was Lu^a^-positive for two of eleven cells.

## Discussion

Antibodies to RBC were observed in 2.2% of Group A samples. Two samples were positive because of daratumumab, the immunization frequency was therefore 2.0% after exclusion of these samples. This is comparable to frequencies found in other studies, e.g. in 0.9% of healthy blood donors [16], and in 2.2% [17] or 2.9% [16] of patients, respectively. Immunization depends largely on whether the subject has received previous transfusions [1]. It can be regarded as a common phenomenon, occurring in 1% to 10% of adult transfusion recipients [18, 19].

In this comparative study of two commercially available red blood cell antibody screening systems, the glass card method was found to be comparable to the gel card system in prospective antibody screening (group A, sensitivities of 90.9% vs. 86.4%, respectively. This confirms roughly the results of a recent Spanish study, which found sensitivities of 87.5% to 100% with samples that were stored up to 72 h at 2–8°C. The reported numbers in that study were higher after the authors included a different group of known positive samples [20]. The primary screening samples group had a low rate of non-negative tests of 0.9%, because 80% of the samples were from healthy blood donors. This impairs the applicability of the results to patient diagnostics, as not only less, but also different antibodies occur in healthy donors.

In a recent prospective study in Thailand, sensitivities of 100% and 85.7% were found for the glass card and gel card system, respectively, for antibodies regarded as significant [21]. Sensitivities for clinically insignificant antibodies were lower: only 53.6% for the glass card and 28.5%–35.7% for two different gel card systems, respectively. However, the Thai researchers also regarded antibodies to Mi^a^, Le^a^, and M, which may cause hemolysis in some cases, as insignificant. These antibodies were found in tube testing, which is otherwise known to be significantly less sensitive [11, 22]. These authors used reagent cells from the Thai Red Cross Society for all methods. If the same low ionic strength buffer was used for all methods, this could have impaired methods that depend on the buffer’s composition [23].

Weisbach et al. [11] found that the glass card and gel card systems have sensitivities of 90.5% and 93.5%, respectively, but they used stored samples for their analysis (comparable to our group B). In our group B, the gel card system was superior to the glass card system for analyzing stored samples with known antibodies. Accordingly, we calculated the positive predictive value (PPV) of the gel card and glass card system to be 95% and 82%, respectively. This difference is plausible as testing included antibodies that were only reactive with enzyme-treated cells and enzyme-treatment is more intense for gel card testing. However, the clinical significance of enzyme-only antibodies is unclear. Antibody screening is generally performed without enzyme-treated cells because the delay required to obtain additional information (e.g., on weak warm-reactive autoantibodies, which are clinically irrelevant) results in a disadvantage that exceeds the potential benefit [24]. In addition, the potential harm of enzyme-only antibodies has long been a matter of debate [25, 26].

Solid-phase testing is established without enzyme-treated cells and has been found to achieve superior sensitivities [11, 27]. This includes testing for prenatal anti-D, which could be detected up to the fifth dilution step in solid-phase testing, while card testing was generally only positive up to the fourth dilution step [28]. The solid-phase system’s superior sensitivity contrasts with its low specificity [12]. Weisbach et al. found inconclusive reactivities, possibly mostly false positive reactions, in 1.38% and 0.13% of samples tested with a solid-phase and gel card system, respectively [11]. Inconsistent results were also found by Quillen et al., who reported for automated solid-phase systems non-repeatability for the detection of antibodies to E, Jk^a^, and Fy^a^ [29]. A recent comparison found unspecified reactions in 7 cases for the solid phase assay compared to 2 for gel card agglutination [12].

In general, studies without sample storage that reflect real world testing were based on a small number of positive samples, which is of questionable significance. The use of stored samples, in contrast, allows investigators to analyze a larger number of samples. Antibodies are usually considered robust to pre-analytical variation. If, for instance, antibodies to Toxoplasma are stored at –20°C, there occurs an approximately 8% decrease in IgG antibodies and a 13% decrease in IgM antibodies, both of which are less than analytical variation [30]. However, compared to antibodies to red cell antigens, antibodies to various viral antigens are more stable in vivo, with half-lives ranging from 50 to 200 years [31]. In contrast, antibodies to red cells have much shorter half-lives of less than 4 years overall [3, 4], and the half-live is dependent from the specificity [3–5]. There are no robust data on red cell antibody decay during storage, but it is likely that storage also affects antibodies differently. As all methods have antibodies that react especially well with the respective method, this bearing damage would effect the performance with stored samples. This effect was apparent with our data. It limits the interpretation of results and the use of statistical data from stored samples.

Batch-to-batch variation of reagent cells was detected as an incidental finding of our study. These fluctuations impacted the test sensitivity for Lu^a^_,_ a less frequent antigen. The corresponding antibody was weakly reactive in many of the stored samples and was only visible in some of the Lu^a^-positive cells. Batch-to-batch variation of reagent cells is possible, as reagent cell donors have different antigen expression levels. Cell storage and preparation methods may also impact reactivity. Therefore, decreased sensitivity with differentiation panels that contain only one instead of two antigen-positive cells is plausible. One group recently published similar findings for Kp^a^, which increases the sensitivity of the test system if included in screening at the cost of having four instead of three cells per sample in the screening [12].

In conclusion, RBC antibody screening sensitivity is important to detect antibodies in evanescence. This favors the use of enzyme-treated cells and to include all important antigens, if blood provision was thereby not impaired.
